# *Toxoplasma gondii* in Aborted Fetuses of Sheep in Ardebil Area, North-West of Iran

**Published:** 2019

**Authors:** Gholamreza SHAHBAZI, Nasser HOGHOOGHI RAD, Rassoul MADANI, Somaieh MATIN, Pedjman MORTAZAVI, Amir Hossein JANGJOU

**Affiliations:** 1. Department of Parasitology and Mycology, Faculty of Specialized Veterinary Science, Islamic Azad University, Science and Research Branch, Tehran, Iran; 2. Department of Clinical Pathology, Faculty of Specialized Veterinary Science, Islamic Azad University, Science and Research Branch, Tehran, Iran; 3. Department of Internal Medicine, Ardabil University of Medical Sciences, Ardabil, Iran; 4. Department of Pathology, Faculty of Specialized Veterinary Science, Islamic Azad University, Science and Research Branch, Tehran, Iran

**Keywords:** *Toxoplasma gondii*, Abortion, Sheep, Iran, Nested PCR

## Abstract

**Background:**

One of the severe complications of toxoplasmosis is the induction of abortion in sheep, goats, and human beings. The rate of abortions related to toxoplasmosis in sheep varies between 10% and 23% in the United States and European countries. In Iran, *Toxoplasma* infection were diagnosed in aborted ovine fetuses between 13.5% and 69% in the brain samples based on PCR. The purpose of this study was to investigate the prevalence of abortions related to *T. gondii* among the aborted fetuses of sheep in Ardabil area, North-West of Iran.

**Methods:**

The brains of 75 aborted sheep fetuses in Ardebil area were investigated with nested PCR method using fragments of the GRA6 gene during lambing seasons in 2014–2015. Meanwhile, thoracic and abdominal fluids of aborted fetuses and 200 serum samples collected from the herds affected by abortion were examined for the existence of anti-*T. gondii* IgG.

**Results:**

For 48 out of the 75 fetal brain samples (64%), the results of nested PCR test were found to be positive. Furthermore, antibodies against *Toxoplasma* was observed in 136 (68%) collected samples of sheep and 21 samples (28%) of fetal fluids.

**Conclusion:**

There is the significant role of *T. gondii* in the abortions of sheep in Ardabil area. Meanwhile, this condition can also be dangerous for human beings because of their consumption of sheep meat.

## Introduction

*Toxoplasma gondii* is an intercellular protozoa which affects warm-blooded vertebrate animals all over the world and can cause abortion in sheep, goats, and human beings (1).

*T. gondii* is identified to be one of the main causes of reproductive disorders in the sheep living in England, New Zeeland, Norway, and some other countries (2–4). When sheep and goats get exposed to this parasite during pregnancy, stillbirth and the birth of weak lambs can take place (5).

In Iran, studies have been conducted on abortions related to toxoplasmosis (6–9). In Ardabil situated in the North-west of Iran, abortions in sheep occur every year, however, no study has attempted to identify factors causing it.

This study aimed to investigate the role of toxoplasmosis in the abortions occurred in the sheep living in this area using nested PCR, serological, and pathological techniques.

## Materials and Methods

Seventy-five *fetuses* with an *estimated* gestational *age* of ≥120 d were collected from 53 different sheep herds during lambing seasons in 2014–2015. For brain samples, upon opening the calvarium, the meninges were dissected using a new disposable scalpel and forceps for each fetus. A sample of brain tissue approximately 1 cm^3^ was excised from of the right cerebral hemisphere and freezed at the temperature of −20 °C for the nested PCR (10). The remainder of the fetal brain was fixed in 10% formalin so as to conduct pathological tests later. Meanwhile, thoracic and abdominal fluids of aborted fetuses collected by sterile syringe. Some sheep from each farm were randomly selected and bled from a jugular vein (200 samples). Collected blood and fetal fluid samples were centrifuged and subsequently stored at −20 °C until used.

### DNA extraction and PCR detection:

*T. gondii* DNA was extracted from aborted fetus brain using the QIA amp DNA mini kit (Qiagen, Courtaboeuf, France) and then stored at −20 °C. Nested primer sets were used for amplifying fragments of the GRA6 gene (11). The external primers were GRA6-F1x (5-ATTTGTGTTTCCGAGCAGGT-3) and GRA6-R1 (GCACCTTCGCTTGTGGTT) producing an amplified product of 546 bp. Internal primers were GRA6-F1 (TTTCCGAGCAGGTGACCT) and GRA6-R1x (TCGCCGAAGAGTTGACATAG) producing an amplified product of 351 bp.

### Serology

Antibodies to *T. gondii* were tested by indirect fluorescent antibody test (IFAT) using *Toxoplasma* IFA slide by Biogene (Iran) and fluorescein-labeled rabbit anti-sheep IgG antibodies (Razi teb co). Firstly, sera were diluted 1:16 for IgG antibodies in phosphate-buffered saline (PBS, 0.1M phosphate, 0.33M NaCl, pH 7.2). Aliquot of 10 μl from each serum was placed on the well of *T. gondii*-slide and incubated in a humidified chamber at 37 °C for 30 min. After washing in PBS, slides were incubated for 30 min at 37 °C with both diluted IgG-FITC (conjugate dilution was 1:500 in 0.2% filtered Evan′s blue dye in PBS). Slides were then washed two times in PBS and examined by using IFA microscope. After this, positive sera were diluted two-fold from 1:32 to 1:64 for IgG antibodies and re-assayed. If positive, further serial twofold dilutions were tested. The highest dilution of serum, which showed a clear fluorescence around the whole tachyzoites, was considered as the final dilution. Positive and negative ovine control sera were included in each slide. For fetal fluid samples, antibody dilution equal to 1/16 were considered as positive titers.

### Histologic examination

For the histologic study, different sections of brain were trimmed and dehydrated through graded alcohols before being embedded in paraffin wax using routine procedures. From each block, four to six sections 4 μm thick were cut semi-serially, deparaffinised, rehydrated and stained with hematoxylineosin and examined by light microscopy.

## Results

### The results of nested PCR

**Fig. 1:**
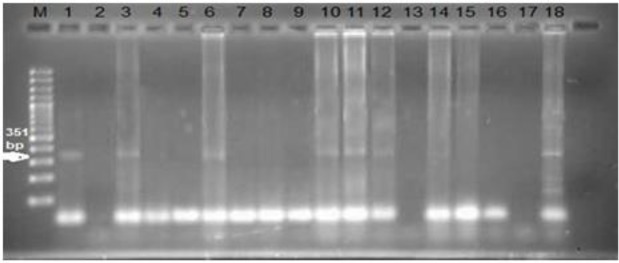
Agarose gel electrophoresis of nested PCR products of tissues samples of aborted fetus with the internal GRA6 primers. Lane M molecular marker (100bp DNA Ladder, Sinaclon Co.), lane 1 *T. gondii* positive control. Lane 2 negative control, Lane 3–18 tissues from aborted fetuses

In the nested-PCR 48 out of 75 (64%) Samples have product bond at the 351 bp on electrophoresis gel with amplifying fragments of the GRA6 gene (Fig. 1).

### IFAT results

Serological diagnosis of toxoplasmosis through IFA test indicated that 136 (68%) out of 200 sheep were seropositive at titers ≥1:64 (Table 1). Moreover, antibody titers ≥ 1:16 were detected in 28% of ovine fetuses.

**Table 1:** Frequency of anti-toxoplasma IFA antibody titers among sheep serum samples in Ardebil area, North-west of Iran

***Titer Result***	***1/16***	***1/32***	***1/64***	***1/128***	***1/256***	***1/512***	***1/1024***
Negative(n)	48	54	64	94	136	172	193
Positive(n)	152	146	136	106	64	28	7
Total(n)	200	200	200	200	200	200	200

### Pathological examinations of aborted fetuses′ brains

**Fig. 2:**
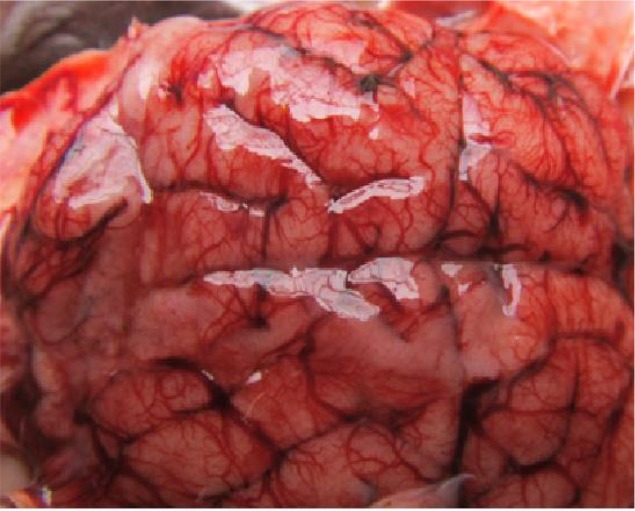
Brain of an aborted sheep fetus. Severe congestion and edema in the brain

Hyperemia and edema were the most common lesions (86%) in the pathological examinations (Fig. 2) and were followed by encephalomalacia (10.3%), meningitis (10.3%), leukomalacia (6.8%), meningoencephalitis (3.4%), demyelination (3.4%), gliosis (3.4%), but *Toxoplasma* cyst wasn′t found in any of the specimens.

## Discussion

The rate of abortions related to toxoplasmosis in sheep varies between 10.6% and 23.1% in the United States and European countries (12, 13).

In this study, *T. gondi* infection was detected in 48 out of 75 aborted fetuses (64%) in the PCR method, also antibodies to *T. gondii* were found in 68% of the studied population that indicates widespread exposure to *T. gondi* in the study area.

Studies performed using PCR on brain samples of aborted fetuses in sheep (6, 7, 14, 15) showed that respectively 54%, 66%, 69% and 13.5% of samples were infected with *Toxoplasma*.

Detecting antibodies against *T. gondii* in aborted ovine fetuses with the IFAT has been used in some studies (13, 16, 17). Antibodies against *T. gondii* detected in 5.2% of aborted ovine fetuses by IFAT (8). In another study in a herd that had abortion, 97.4% samples of aborted lambs had antibodies against *Toxoplasma* (18).

In the present study, positive results for anti-*T. gondii* antibodies in fetal fluids compared to the positive results of PCR in the brain samples of the fetus were much low. One of the reasons explained is that in the acute phase of *T. gondii* infection when the fetal death occurs in only few days, the progression of the disease is so rapid that the patient′s immune system has not enough time to produce detectable antibodies (19).

In a study similar to the results of our study analyzed the human aborted fetuses for which the PCR test was positive using immunohistochemistry (IHC) method and did not observe any cyst or tachyzoite in them. The reason for this might be the low number of parasites in the aborted tissues (20).

The results of this study showed that the prevalence of toxoplasmosis in Ardabil in compared with other studies in Iran (21–23) is high. This finding confirms the studies in this area (24). It is possible that extensive and uncontrolled pasture in this region has provided the conditions for widespread sheep contamination. High seroprevalence levels in sheep were reported in the USA (25), Turkey (26), Serbia (27), Scotland (28) and France (29). These differences can be attributed mainly to some epidemiologic factors such as age of the animals (25, 28–32) traditional sheep farming, wide distribution of stray cats and high relative humidity which can effect on the viability and sporulation of oocysts (33).

Although the role of *T. gondii* has been identified and reported in small ruminants all over the world (7, 8,13), the findings of the present study showed the role of this parasite in the abortions of sheep in the study area.

## Conclusion

*T. gondii* may be one of the potential agents in causing significant rate of sheep abortion in the Ardabil area. Moreover, this situation may somehow demonstrate health danger for the human inhabitants who ordinarily consume sheep meat.
